# Supplementary material for: Structural Basis of RICs Iron Donation for Iron-Sulfur Cluster Biogenesis
Source: Front Microbiol. 2021 Apr 29;12:670681. doi: 10.3389/fmicb.2021.670681 (PMC8117158; doi:10.3389/fmicb.2021.670681)
Supplement: Supplementary file 1 [file Data_Sheet_1.pdf]

frontiers

Supplementary Material

1. Supplementary Figures

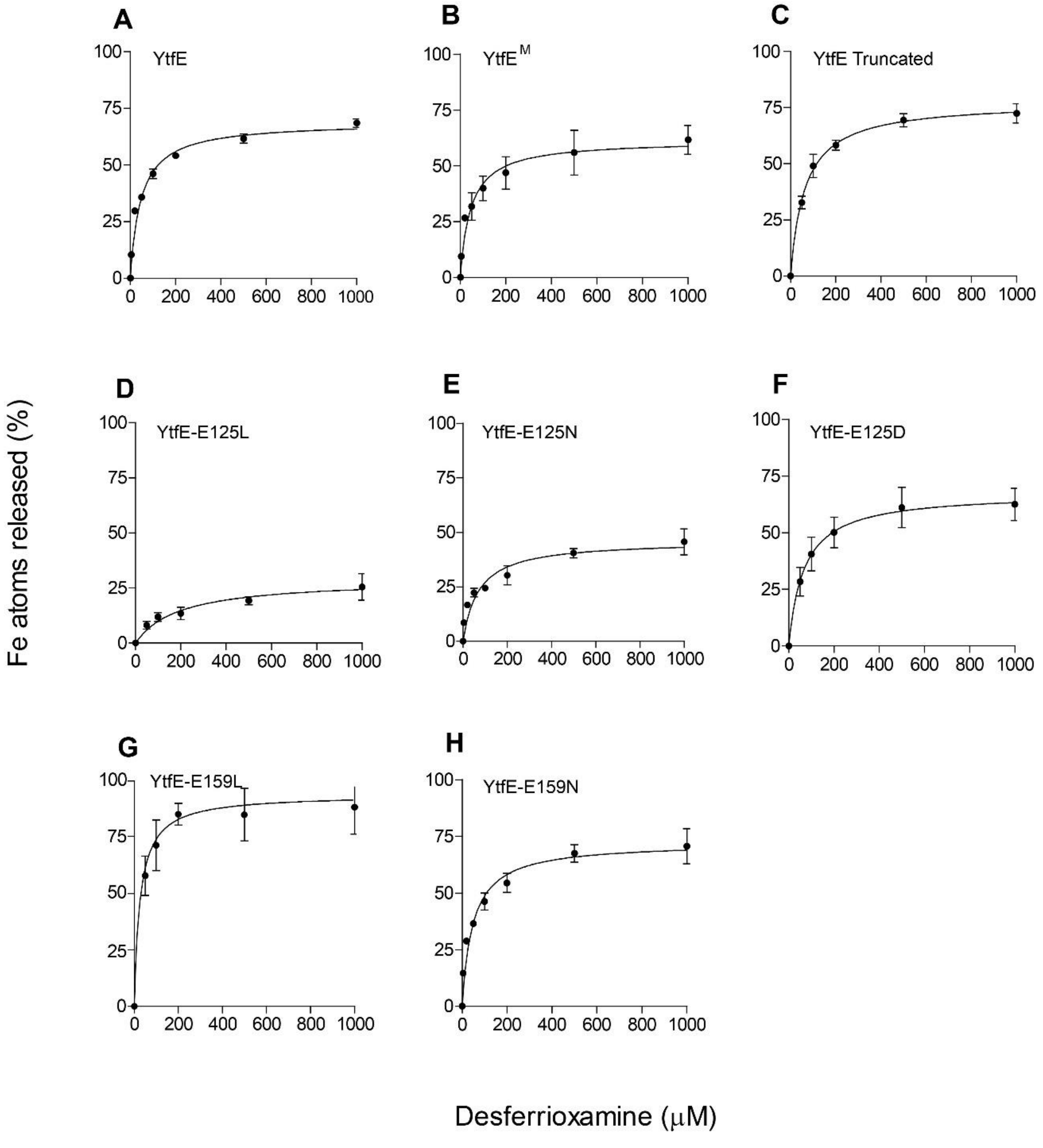

Supplementary Figure 1: Ferric iron release from YtfE in the presence of desferrioxamine.

As-isolated proteins (~25 µM) were incubated with 0-1000 µM desferrioxamine under aerobic conditions. The UV-visible spectra were acquired after 24 h and the absorbance at 420 nm of the complex Fe (III)-desferrioxamine, was used to determine the percentage of iron released, as indicated in the Methods section. Assays were performed with at least two independent protein samples.

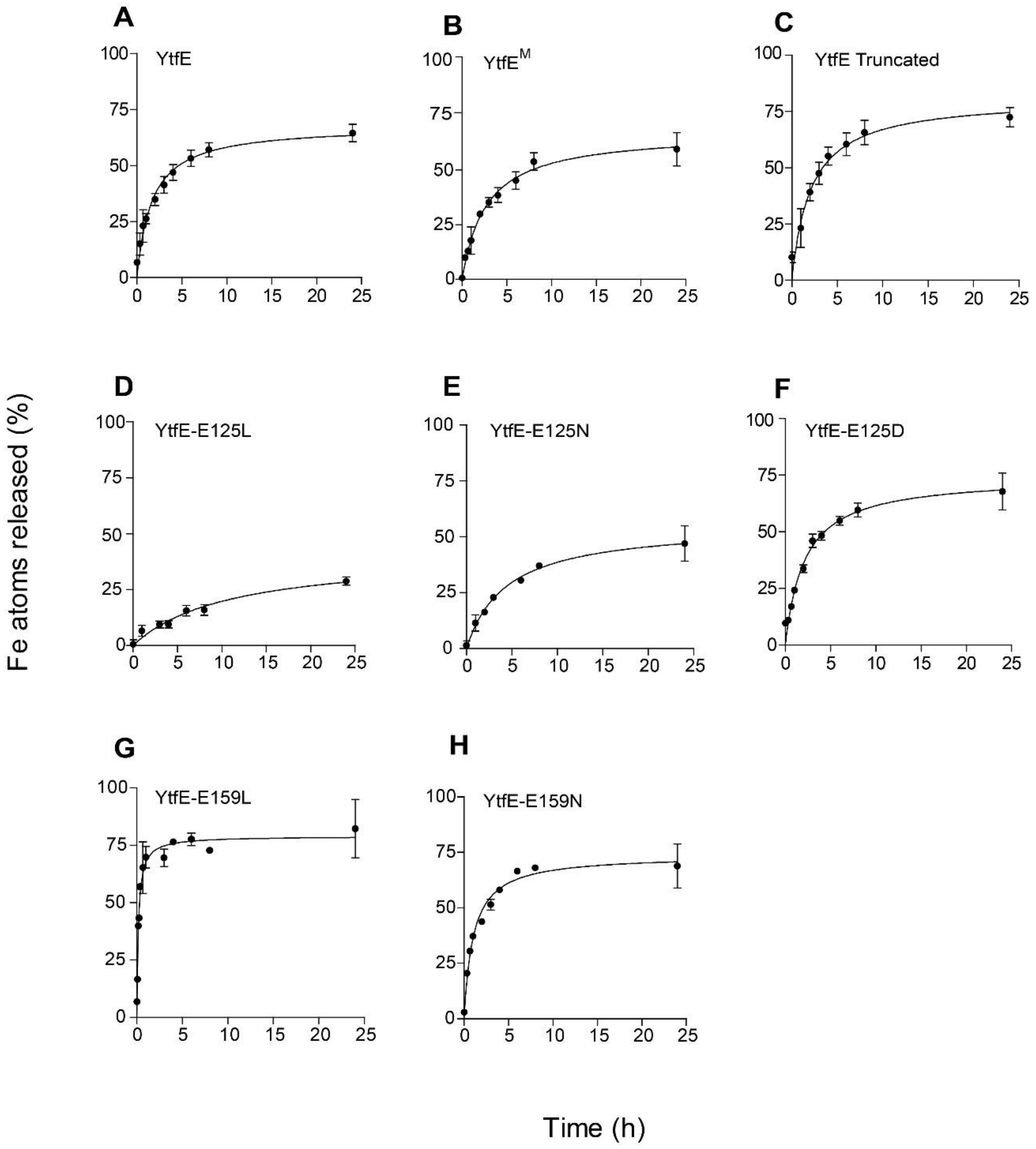

Supplementary Figure 2: Monitoring the rate of ferric iron release from YtfE mutants over time. As-isolated proteins (~25 μM) were incubated under aerobic conditions with 1000 μM desferrioxamine. The release of iron was monitored by acquiring spectra over time (0-24 h). The percentage of iron released was determined using the absorbance at 420 nm of the complex Fe (III)-desferrioxamine. The rate of iron release was determined by the slope of a linear fit to the data points taken within the first hours of the reaction. The assays were performed with at least two independent protein samples.

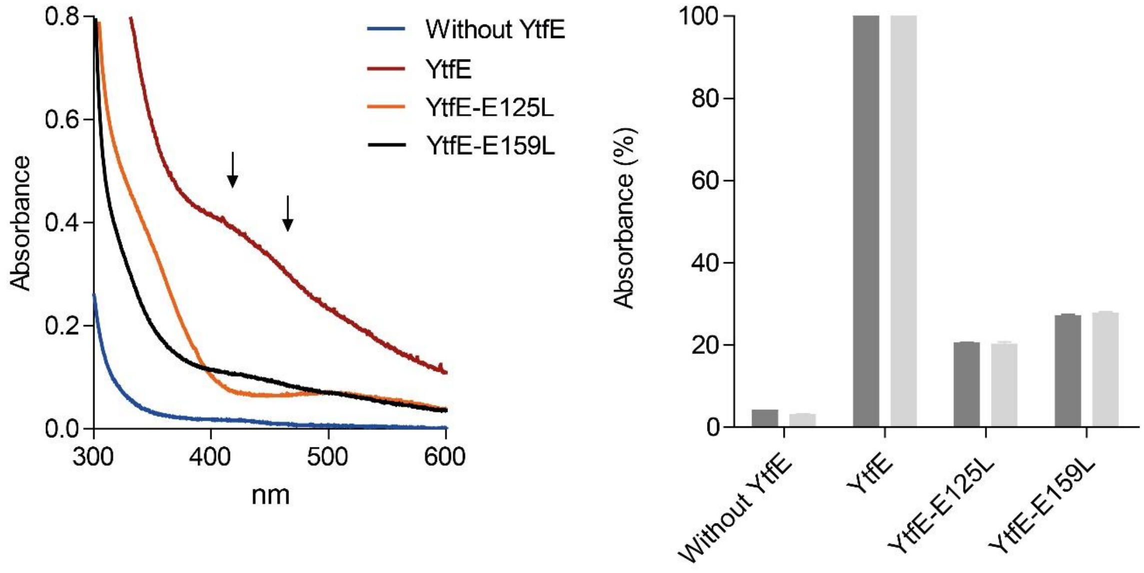

Supplementary Figure 3: Reconstitution of Fe-S centre in apo-IscU using YtfE. Reaction mixtures containing YtfE (wild-type or mutant proteins), IscS and DTT were incubated for 1h. The formation of a mixture of $[2Fe\text{-}2S]^{2+/1+}$ and $[4Fe\text{-}4S]^{2+/1+}$ clusters in IscU is shown by the appearance of two bands at ~456 nm and ~410 nm that are only observed in the UV-visible spectrum of the reaction done with wild-type YtfE (represented in red on the left panel). For each reaction mixture, the right panel shows the absorbance at 456 nm (light gray) and 410 nm (dark gray) relative to the absorbance of the same bands measured for the reaction done with wild-type YtfE.

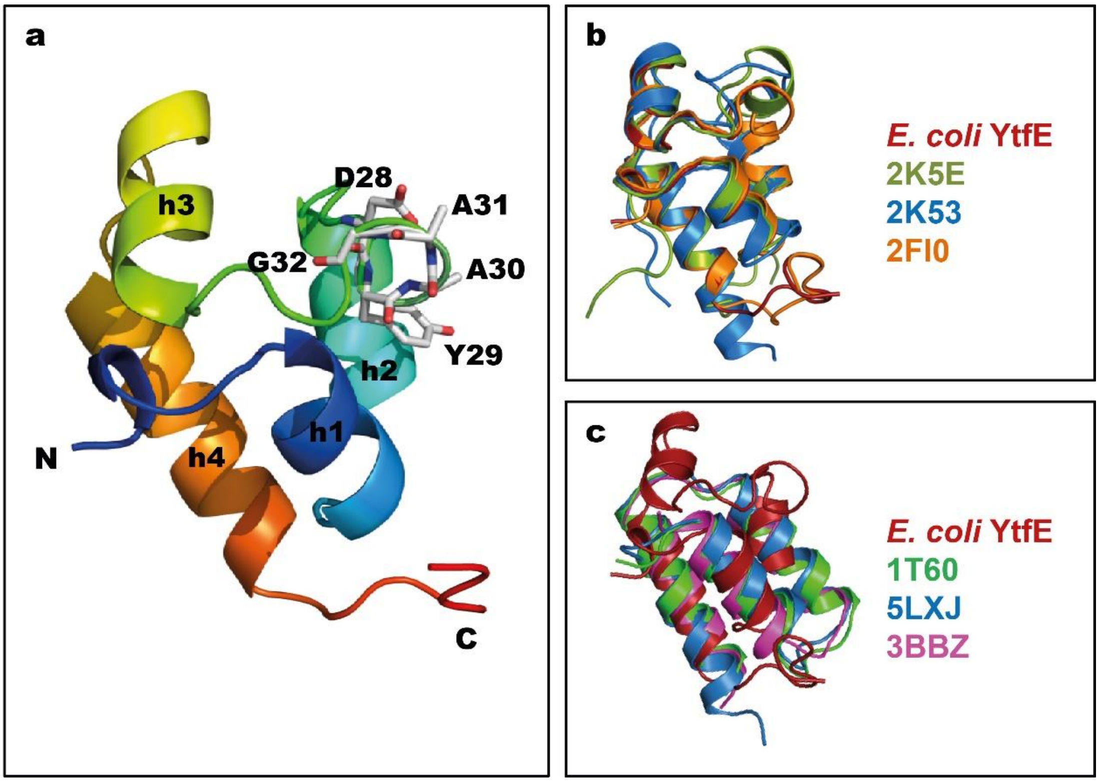

Supplementary Figure 4: Overall structure of domain A in *E. coli* YtfE^{M}. a, Cartoon representation of domain A, rainbow coloured from dark blue at the N-terminal (N) to red at the C-terminal (C). The residues from the DYAAG motif are represented as sticks. The residues A30 and A31 correspond to the two cysteines C30 and C31 in the wild-type protein. **b,** Cartoon representation of the structural superposition of *E. coli* YtfE^{M}-E125L with uncharacterized bacterial protein structures with PDB codes 2K5E (*Methanocaldococcus jannaschii*), 2K53 (*Clostridium thermocellum*) and 2FI0 (*Streptococcus pneumoniae* TIGR4), that present high structural homology but low sequence similarity. **c,** Cartoon representation of the structural superposition of *E. coli* YtfE^{M}-E125L with the structures with PDB codes 1T6O (nucleocapsid-binding domain of the measles virus P protein), 5LXJ X (domain of *Peste des Petits Ruminants* phosphoprotein) and 3BBZ (nucleocapsid-binding domain from the mumps virus phosphoprotein). These three proteins lack the region between helices 2 and 4.

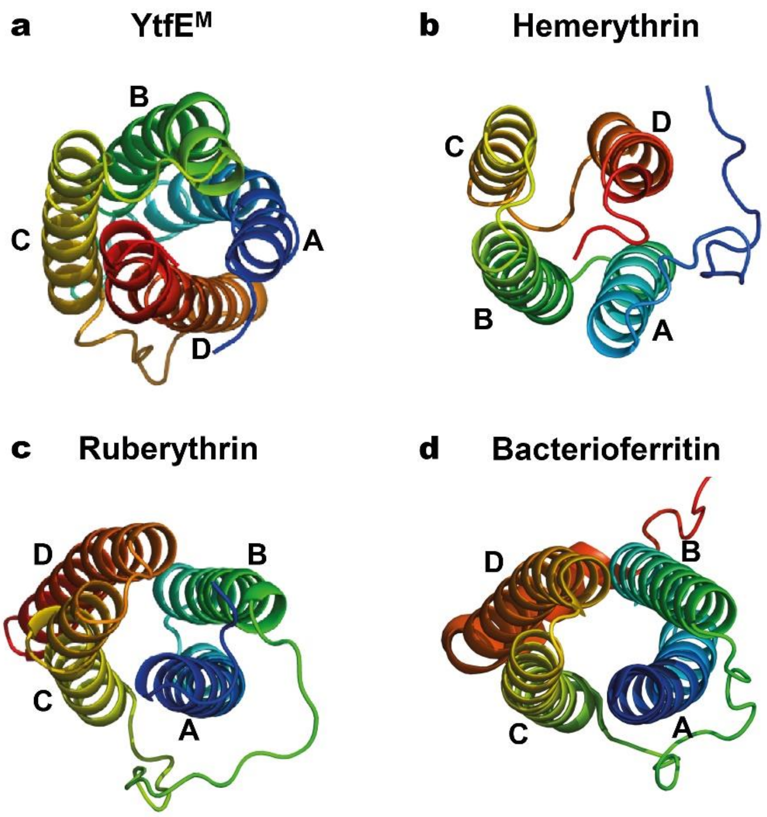

Supplementary Figure 5: Arrangement of the four-helix bundles in YtfE, rubrerythrin, hemerythrin and ferritin-like proteins. The helices are coloured from N-terminal in blue to C-terminal in red. **a.** YtfE^M (PDB code 7BHA) is a left-handed four-helix bundle; **b.** Hemerythrin (PDB code 2MRH) is a right-handed four-helix bundle; **c.** Rubrerythrin (PDB code 1RYT), and **d.** Bacterioferritin (PDB code 1NFV) both starting as left-handed and become right-handed four-helix bundles.

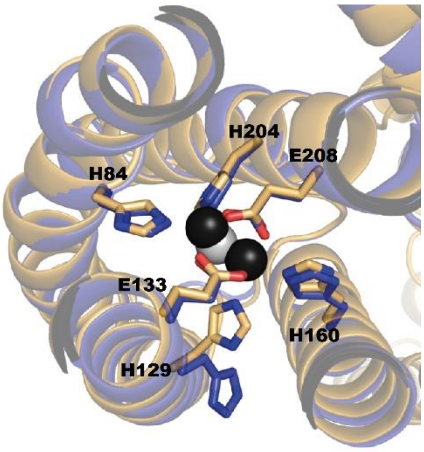

Supplementary Figure 6: Superposition of the di-iron centre between YtfE^{M} and YtfE^{M}-E159L. Detail of YtfE^{M}-E159L (represented in blue) showing that H129 assumes a different side chain conformation from that in YtfE^{M} (shown in orange). The mononuclear Fe centre of YtfE^{M}-E159L is shown as a grey sphere, the di-iron centre of YtfE^{M} is represented as black spheres and the coordinating residues H84, H160, H204, E208 and E133 are indicated as sticks.

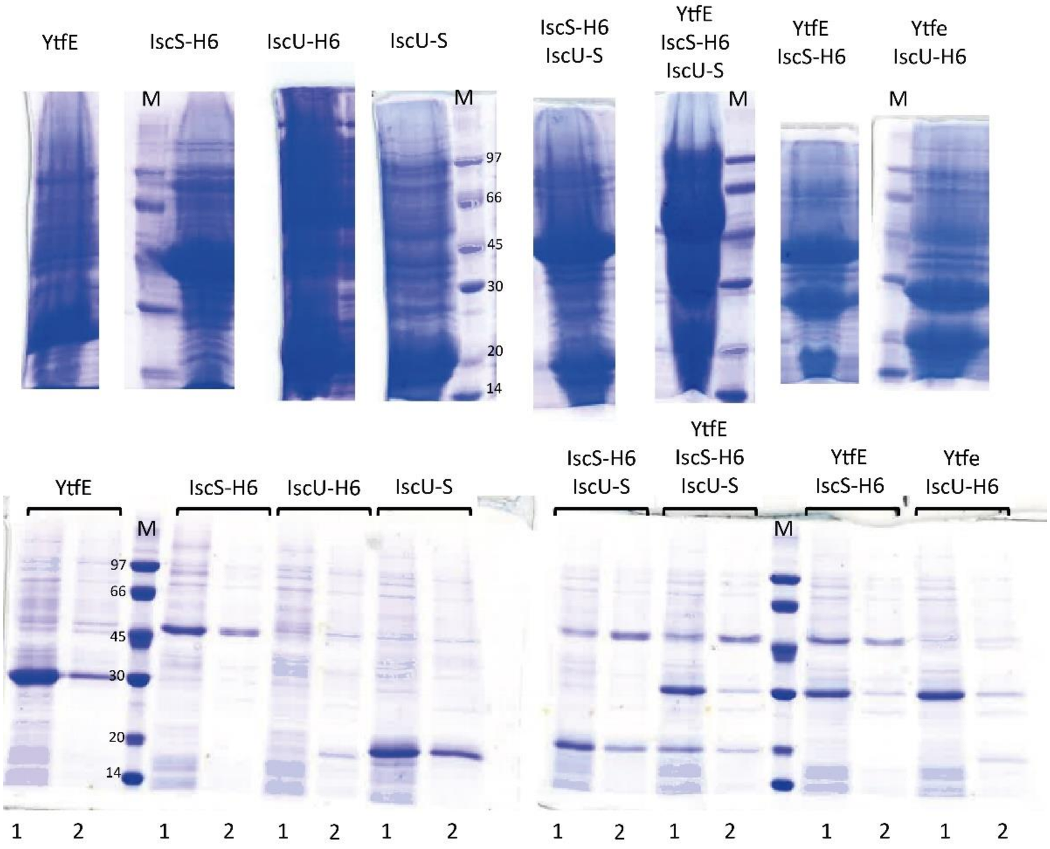

Supplementary Figure 7: SDS-PAGE of samples used in the pulldown assays. Upper panel shows cell extracts immediately before loaded into the column. Lower panel exhibits samples collected at different stages: lanes 1, after washing the column with 15 mL of binding buffer, and lanes 2 after column washing with 25 mL of buffer containing 60 mM of imidazole.

2. Supplementary Tables

Supplementary Table 1: Data collection and processing statistics

Data Set	*E. coli* YtfE^M	*E. coli* YtfE^M-E159L	*E. coli* YtfE^M-E125L
**Beamline**	ALBA XALOC	ESRF ID30A-3	Diamond I04
**Detector**	Pilatus 6M	Eiger X 4M	Eiger2 XE 16M
**Wavelength (Å)**	1.7314	0.96770	0.97950
**Space group**	$P2_1$	$P2_1$	$P2_1$
**Unit-cell parameters (Å, °)**	a=60.06, b=50.65, c=87.85, β=100.54	a=86.81, b=51.42, c=54.96 β= 90.103	a=59.79, b=49.60, c=87.81, β=100.14
**Data Processing**	XDS/POINTLESS/AIMLESS	AutoPROC/XDS /POINTLESS/STARANISO/AIMLESS	XDS/POINTLESS/AIMLESS
**Resolution limits of ellipsoid fitted to resolution cut-off surface (Å)**	-	2.89, 2.22, 3.23	-
**Resolution range (Å)**	59.05 - 2.19 (2.23 - 2.19)	86.81 – 2.36 (2.64 – 2.36)	86.44 – 1.87 (1.98 – 1.87)
**N° of observations**	149792 (2690)	43151 (2152)	155605 (7101)
**Unique reflections**	23568 (549)	11106 (556)	41865 (2086)
**<I/σ(I)>**	9.8 (1.6)	10.2 (1.4)	9.7 (0.9)
R_{merge} [a]	0.147 (1.548)	0.088 (0.828)	0.082 (1.232)
R_{meas} [b]	0.160 (1.731)	0.102 (0.959)	0.096 (1.457)
R_{pim} [c]	0.062 (0.759)	0.052 (0.479)	0.049 (0.767)
**CC 1/2**	0.985 (0.354)	0.998 (0.568)	0.999 (0.360)
**Completeness, spherical (%)**	87 (40.2)	55.1 (9.6)	99.8 (99.4)
**Completeness, ellipsoidal (%)**	-	86.8 (52.7)	-
**Multiplicity**	6.4 (4.9)	3.9 (3.9)	3.7 (3.4)
**Wilson B-factor (Å^2)**	37.8	47.6	30.8
**No. of molecules in a.u.**	2	2	2
**V_m (Å^3 Da^{-1})**	2.65	2.47	3.03
**Estimated solvent content (%)**	53.7	50.2	59.4

Values in parentheses are for the highest resolution shell. [a] Merging *R*-factor, $R_{merge} = (\Sigma_{hkl} \Sigma_i |I_i(hkl) - \langle I(hkl)\rangle|) / (\Sigma_{hkl} \Sigma_i I(hkl)) \times 100$ %, where $I_i(hkl)$ is the intensity measured for each unique Bragg reflection with indexes (hkl), $\langle I(hkl)\rangle$ is the average intensity for multiple measurements of this reflection. [b] Multiplicity independent *R*-factor, $R\text{-meas} = \Sigma_{hkl} [N_{hkl}/(N_{hkl}-1)]^{1/2} \Sigma_i |I_i(hkl) - \langle I(hkl)\rangle| / \Sigma_{hkl} \Sigma_i I(hkl)) \times 100$ %. [c] Precision independent *R*-factor, $R_{pim} = \Sigma_{hkl} [1/(N_{hkl}-1)]^{1/2} \Sigma_i |I_i(hkl) - \langle I(hkl) \rangle| / \Sigma_i I_i(hkl) \times 100$ %, where $I_i(hkl)$ is the observed intensity, $\langle I(hkl)\rangle$ is the average intensity of multiple observations from symmetry-related reflections, and N_{hkl} is their multiplicity(Diederichs and Karplus, 1997).

Supplementary Table 2: Structure refinement statistics

Data Set	*E. coli* YtfE^{M}	*E. coli* YtfE^{M}-E159L	*E. coli* YtfE^{M}-E125L
Resolution limits (Å)	59.05 – 2.19 (2.23-2.19)	46.40 – 2.36 (2.71 – 2.36)	53.20 – 1.87 (1.92 - 1.87)
R-factor [a]	0.212 (0.332)	0.2516 (0.3787)	0.183 (0.358)
nr. Reflections [b]	43180 (1256) [c]	10610 (771)	39805 (2633)
Free *R*-factor [d]	0.250 (0.349)	0.2962 (0.3362)	0.211 (0.392)
nr. Reflections [b]	2235 (73)	483 (30)	2037 (113)
Coordinate error estimate (Å) [e]	0.28	0.36	0.29
**Model completeness and composition**			
Regions omitted	1A, 1B-8B	1A-4A, 1B	1A, 1B
N° molecules in asymmetric unit	2	2	2
Non-hydrogen protein atoms	3512	3446	3523
Iron ions	4	2	4
Solvent molecules	83	12	323
**Mean B values (Å^2)** [f]			
Protein chain A	48.4	72.6	35.1
Protein chain B	67.6	76.3	59.9
Fe atoms	66.9	49.4	56.0
Solvent molecules	50.6	22.2	45.1
**Model r.m.s. deviations from ideality**			
Bond lengths (Å)	0.002	0.002	0.007
Bond angles (°)	0.464	0.445	0.806
Chiral centers (Å^3)	0.033	0.034	0.044
Planar groups (Å)	0.003	0.003	0.006
**Model validation** [g]			
% Ramachandran outliers	0.23	1.16	0
% Ramachandran favoured	95.78	92.11	98.16
% Rotamer outliers	0	0.27	0.79
C$^{\beta}$ outliers	0	0	0
Clash score	2.19	4.51	1.68
**PDB accession ID**	**7BHA**	**7BHB**	**7BHC**

Values in parentheses are for the highest resolution shell. [a] R-factor = $\Sigma_{hkl} ||F_o|-|F_c|| / \Sigma_{hkl} |F_o|$, where $|F_o|$ and $|F_c|$ are the observed and calculated structure factor amplitudes, respectively; [b] no $\sigma(F_o)$ cutoff; [c] the Bijvoet mates were treated as separate observations in the refinement; [d] cross-validation R-factor computed from a randomly chosen subset of 5% of the total number of reflections that was not used in the refinement; [e] Maximum-likelihood estimate; [f] Calculated from isotropic or equivalent isotropic B-values; [g] Calculated with MOLPROBITY(Chen et al., 2010).

Supplementary Table 3: B-values

	YtfE^M	YtfE^M-E159L	YtfE^M-E125L
**Chain A**	52.6	83.5	40.3
**Chain B**	73.8	88.4	67.6
**Domain A, chain A**	70.6	126.0	42.1
**Domain A, chain B**	123.4	132.2	95.4
**Domain B, chain A**	44.5	65.5	39.5
**Domain B, chain B**	53.6	68.5	55.3

Supplementary Table 4: Distances (Å) between irons (Fe1 and Fe2) and the ligand atoms from coordinating residues or other ligands.

		YtfE^M	YtfE^M-E125L			YtfE^M-E159L
**Fe1**	**H84 N$^{\varepsilon 2}$**	2.19 Å	2.15 Å	**Fe1**	**H84 N$^{\varepsilon 2}$**	2.72 Å
	**H204 N$^{\varepsilon 2}$**	2.16Å	2.17 Å		**H204 N$^{\varepsilon 2}$**	2.35 Å
	**E133 O$^{\varepsilon 1}$**	2.11 Å	2.10 Å		**E133 O$^{\varepsilon 2}$1**	1.97 Å
	**E208 O$^{\varepsilon 2}$**	2.06 Å	1.96 Å		**E133 O$^{\varepsilon 2}$**	2.63Å
	**O**	2.02 Å	1.58 Å		**E208 O$^{\varepsilon 1}$**	2.53 Å
**Fe2**	**H129 N$^{\varepsilon 2}$**	2.13 Å	2.14 Å		**E208 O$^{\varepsilon 2}$**	1.91 Å
	**H160 N$^{\varepsilon 2}$**	2.16 Å	2.14 Å		**H_2O**	3.17 Å
	**E133 O$^{\varepsilon 2}$**	2.00 Å	1.94 Å			
	**E208 O$^{\varepsilon 1}$**	2.07 Å	2.16 Å			
	**O**	2.05 Å	2.04 Å			

Supplementary Table 5: Oligonucleotides used in this work

Primer	Sequence
Site-directed mutagenesis	
C3031A sense	5’-TATGATATGGATTACGCCGCTGGCGGTAAGCAG
C3031A anti	5’-CTGCTTACCGCCAGCGGCGTAATCCATATCATA
E125L sense	5´-CATGCTGCATGAACTGCTTTCCAG
E125L anti	5’-GCTGGAAAGCAGTTCATGCAGCATG
E125N sense	5’-CATGCTGCATGAAAATCTTTCCAGCC
E125N anti	5’-GTGGCTGGAAAGATTTTCATGCAGC
E125D sense	5’-CATGCTGCATGAAGATCTTTCCAG
E125D anti	5’-GCTGGAAAGATCTTCATGCAGCATG
E159N sense	5’-GTAATGGAAAGCAATCACGATGAAGC
E159N anti	5’-GCTTCATCGTGATTGCTTTCCATTAC
Gene cloning	
IscU(T18)Fw	5’-GAATCAGGGGATCCTATAATGGC
IscU(T18)Rv	5’-GAAGCAAAGGTACCGTTGAGGTTT
IscS(T18)Fw	5’-GAGTGATGGATCCAGTTTATAGAG
IscS(T18)Rv	5’-GGCTCATCAGGTACCCGGTATCG
YtfE(flag)Fw	5’-TGAGGTATCACATATGGCTTATCGC
YtfE(flag)Rv	5’-CAGCTTTTGGTACCCTCACCCGCC
IscS(pacyc)Fw	5’-GTACGGAGTGGATCCAGCAATGAAAT
IscS(pacyc)Rv	5’-GCCATTATAAGCTTTCCTGATTCCG
IscU(pacyc)Fw	5’-GAATCAGGGGATCCTATAATGGCTT
IscU(pacyc)Rv	5’-GTAATCGACATAACCAAGCTTCAACTC
IscU(pacyc-S)Fw	5’-CAGGAGAATTTCATATGGCTTACAG
IscU(pacyc-S)Rv	5’-CCTCAACTCGAGTTTTGCTTCACG
IscS(pet11)Fw	5’-GTACGGAGTCTCGAGAGCAATGAAAAT
IscS(pet11)Rv	5’-GTAAGCCATTATGGATCCTCCTGATTC
IscS(pbad)Fw	5’-AGTTTATAGAGCCATGGAATTACCGAT
IscS(pbad)Rv	5’-ATTCCGATACCGACGTCTGATGAGC
IscU(pet11)Fw	5’-CGGACTCGAGAGAATTTATAATGGCT
IscU(pet11)Rv	5’-GTAATCGACGGATCCAAACCTCAAC
IscU(pbad)Fw	5’-GAATTTACCATGGCTTACAGCGAAAAAGTTATC
IscU(pbad)Rv	5’-CAAACCTCAACGACGTCTTTGCTTCAC

Supplementary Table 6: Plasmids and strains used in this work

Plasmids	Description	Reference/ Source
pET-YtfE	Vector for expression	(Justino et al., 2006)
pET- YtfE^{M} (C30AC31A)	Vector for expression	This work
pET- YtfE^{M}-E125L	Vector for expression	This work
pET- YtfE^{M}-E159L	Vector for expression	This work
pET-YtfE-E125L	Vector for expression	This work
pET-YtfE-E125N	Vector for expression	This work
pET-YtfE-E125D	Vector for expression	This work
pET-YtfE-E159L	Vector for expression	(Nobre et al., 2015)
pET-YtfE-E159N	Vector for expression	This work
pET-YtfE^{Truncated}	Vector for expression	(Nobre et al., 2015)
pQE60-IscU-(His)6	Vector for expression	Laboratory stock
pQE30-(His)6-IscS	Vector for expression	Laboratory stock
pKT25 / pKTN25	Vector that allows construction of fusions at the N-terminal/C-terminal of T25 fragment (amino acids 1–224 of CyaA)	Euromedex
pUT18 / pUT18C	Vector that allows construction of in-frame fusions at the N-terminal/C-terminal of T18 fragment (amino acids 225–399 of CyaA)	Euromedex
pKT25-YtfE	YtfE fused to T25 fragment in C-terminal	(Silva et al., 2018)
pKTN25-YtfE	YtfE fused to T25 fragment in N-terminal	(Silva et al., 2018)
pUT18-IscU	IscU fused to T18 fragment in N-terminal	This work
pUT18-IscS	IscS fused to T18 fragment in N-terminal	This work
pUT18C-IscS	IscS fused to T18 fragment in C-terminal	This work
pFLAG-YtfE	Vector for expression	This work
pACYC-(His)6-IscS	Vector for expression	This work
pACYC-(His)6-IscU	Vector for expression	This work
pACYC-IscU-(S-tag)	Vector for expression	This work
pACYC-(His)6-IscS-IscU-(S-tag)	Vector for double expression	This work
pET11a-link-N-GFP	Vector for expression of fusions with N-terminal fragment of GFP	Addgene
pMRBAD-link-C-GFP	Vector for expression of fusions with C-terminal fragment of GFP	Addgene

pET11a-YtfE-N-GFP	YtfE fused to N-terminal GFP fragment	(Silva et al., 2018)
pMRBAD-YtfE-C-GFP	YtfE fused to C-terminal GFP fragment	(Silva et al., 2018)
pET11a-IscU-N-GFP	IscU fused to N-terminal GFP fragment	This work
pMRBAD-IscU-C-GFP	IscU fused to C-terminal GFP fragment	This work
pET11a-IscS-N-GFP	IscS fused to N-terminal GFP fragment	This work
pMRBAD-IscS-C-GFP	IscS fused to C-terminal GFP fragment	This work

***E. coli* Strain**	**Description**	**Source**
XL1-Blue	recA1 endA1 gyrA96 thi-1 hsdR17 supE44 relA1 lac [F´ proAB lacIqZΔM15 Tn10 (Tetr)]	Agilent
BL21 DE3 Gold	B F- ompT hsdS(rB– mB–) dcm+ Tetr gal λ(DE3) endA The	Agilent
DHM1	F-, cya-854, recA1, endA1, gyrA96 (Nal^r), thi1, hsdR17, spoT1, rfbD1, glnV44(AS)	Euromedex
M15	M15/F'M15 (DZ291)	Quiagen

References

Chen, V. B., Arendall, W. B., Headd, J. J., Keedy, D. A., Immormino, R. M., Kapral, G. J., et al. (2010). MolProbity: All-atom structure validation for macromolecular crystallography. *Acta Crystallogr. Sect. D Biol. Crystallogr.* 66, 12–21. doi:10.1107/S0907444909042073.

Diederichs, K., and Karplus, P. A. (1997). Improved R-factors for diffraction data analysis in macromolecular crystallography. *Nat. Struct. Biol.* 4, 269–275. doi:10.1038/nsb0497-269.

Justino, M. C., Almeida, C. C., Gonçalves, V. L., Teixeira, M., and Saraiva, L. M. (2006). *Escherichia coli* YtfE is a di-iron protein with an important function in assembly of iron-sulphur clusters. *FEMS Microbiol. Lett.* 257, 278–84. doi:10.1111/j.1574-6968.2006.00179.x.

Nobre, L. S., Lousa, D., Pacheco, I., Soares, C. M., Teixeira, M., and Saraiva, L. M. (2015). Insights into the structure of the diiron site of RIC from *Escherichia coli*. *FEBS Lett.* 589, 426–431. doi:10.1016/j.febslet.2014.12.028.

Silva, L. S. O., Baptista, J. M., Batley, C., Andrews, S. C., and Saraiva, L. M. (2018). The di-iron RIC protein (YtfE) of *Escherichia coli* interacts with the DNA-binding protein from starved cells (Dps) to diminish RIC protein-mediated redox stress. *J. Bacteriol.* 200. doi:10.1128/JB.00527-18.
